# Supplementary material for: CX-5461 Preferentially Induces Top2α-Dependent DNA Breaks at Ribosomal DNA Loci
Source: Biomedicines. 2024 Jul 8;12(7):1514. doi: 10.3390/biomedicines12071514 (PMC11275095; doi:10.3390/biomedicines12071514)
Supplement: Supplementary file 1 [file biomedicines-12-01514-s001.zip › biomedicines-3036110-supplementary.pdf]

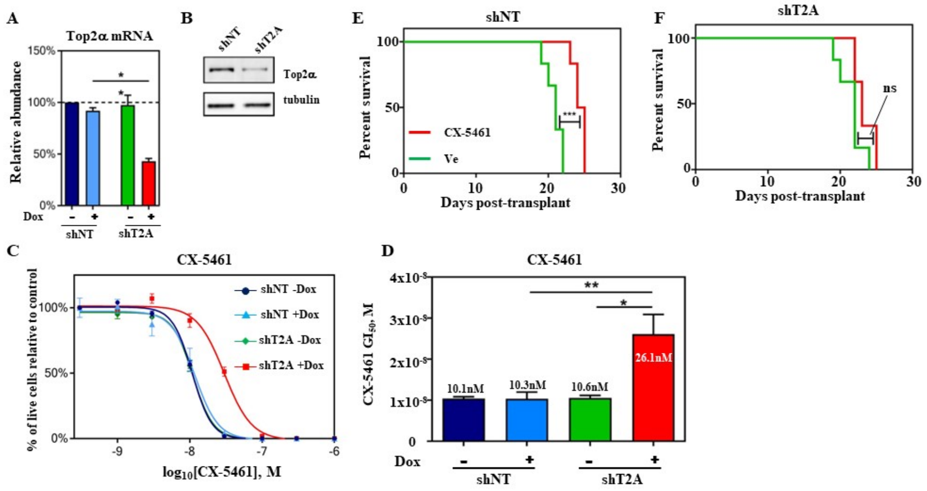

Figure S1. Top2α mediates resistance to CX-5461 in cellular models. (**A**) Top2α mRNA levels in shNT and shT2A cells ± Doxycycline. Basal Top2α mRNA abundance was measured by real-time quantitative PCR (qPCR) and normalized to B2M expression. Error bars represent standard deviation (* $p < 0.05$, $n = 3$). (**B**) Top2α protein expression in shNT and shT2A cells ± Doxycycline. Representative Western blot of basal Top2α protein. (**C**) CX-5461 in vitro dose response assay. The shNT and shT2A cells were treated with 0.3 nM–1 µM CX-5461 for 24 h in triplicate +/– pre-treatment with doxycycline for 48 h and live cell number was counted by volumetric FACS of PI negative cells. The line-of-best-fit was plotted and concentration of the drug inhibiting 50% of growth (GI_{50}) were calculated using GraphPad Prism 10. (**D**) CX-5461 GI_{50} determined from C and plotted as bar graph. (**E**,**F**) Kaplan-Meier curves of doxycycline-induced shNT (**E**) and shT2A cells (**F**) +/– CX- 5461 treatment. Immunodeficient NOD.Cg-*Prkdc*^{scid} *Il2rg*^{tm1Wjl}/SzJ (NSG) mice were transplanted with Eµ-*Myc* shNT and shT2A cells and were fed doxycycline in the food and water to induce hairpin expression in the tumor cells. Upon tumor cell engraftment, mice were treated with 35 mg/kg CX-5461 Q3D. The median survival of mice for the shNT (right panel) and shT2A (left panel) cells was three days and one day, respectively (** $p < 0.01$, n.s. = not significant, n = 6/group).

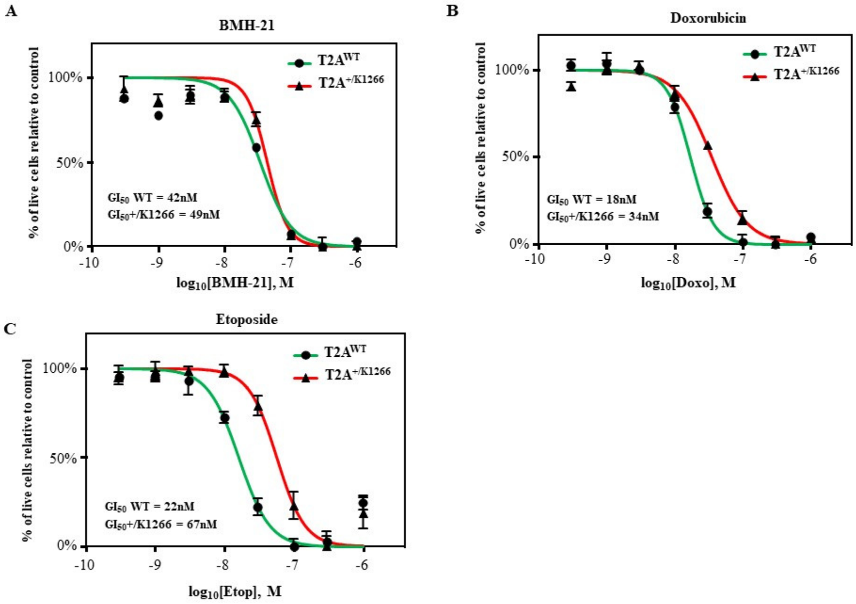

Figure S2. The level of Top2α determines cells sensitivity to Top2 poisons, but not to Pol I inhibitors. (**A**–**C**) In vitro dose response assays. T2A^{WT} and T2A$^{+/K1266}$ cells were treated with varying concentrations of BMH-21 (**A**), doxorubicin (**B**), and etoposide (**C**) for 24 h in triplicate. For (**A**), live cell number was counted by volumetric FACS of propidium iodide (PI) negative cells. For (**B**,**C**), cell viability was measured by 570 nm absorption after Alamar blue treatment. Results were normalized by calibrating the readout from the vehicle-treated cells as 100% and the lowest readout as 0%, and a line-of-best-fit was plotted and concentration of the drug inhibiting 50% of growth (GI_{50}) were calculated using GraphPad Prism 10.

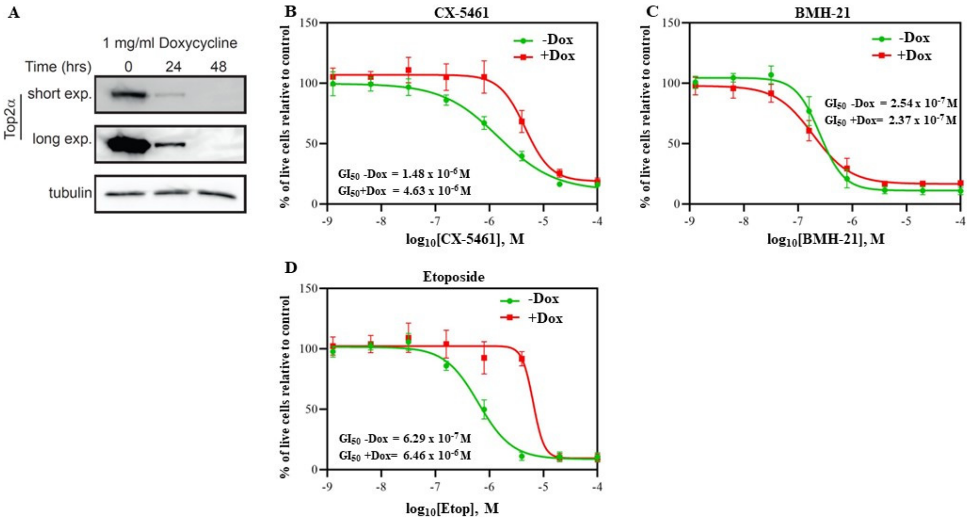

Figure S3. Top2α depletion de-sensitize cells to CX-5461 and Top2 poisons, but not to other Pol I inhibitors. (**A**) Representative Western blot of Top2α protein. HTETOP cells were grown for 24 and 48 h in the presence of 1 mg/mL Doxycycline (Dox) and cell lysates at different time points after Dox addition were analyzed by Western blot. (**B**–**D**) *In vitro* dose response assays. HTETOP cells were grown for 24 h either untreated (-Dox) or in the presence of 1 mg/mL Doxycycline (+Dox) and then treated with varying concentrations of CX-5461 (**B**), BMH-21 (**C**), and etoposide (**D**) in triplicate. Cell viability was measured using PrestoBlue. Results were normalized to vehicle-treated cells (set 100%), a line-of-best-fit was plotted and concentration of the drug inhibiting 50% of growth (GI_{50}) were calculated using GraphPad Prism 10.

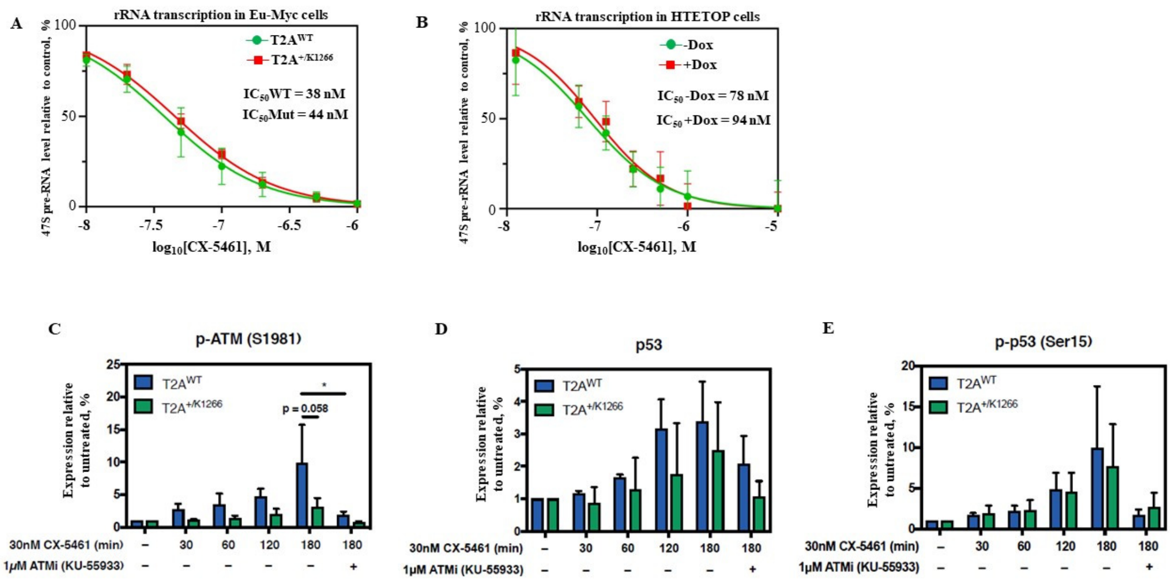

Figure S4. Top2α determines activation of DDR pathways in CX-5461 treated cells but not the ability of CX-5461 to inhibit Pol I transcription. (**A**) Quantification of experiment shown in Figure 2A. Error bars represent standard deviation (n = 3). Results were normalized by calibrating the readout from the vehicle-treated cells as 100%, a line-of-best-fit was plotted and concentration of the drug inhibiting 50% of rRNA synthesis (IC_{50}) were calculated using GraphPad Prism 10. (**B**) Top IIα depletion has no detectable effect on inhibition of Pol I transcription by CX-5461. HTETOP cells were grown for 24 hours either untreated (-Dox) or in the presence of 1 mg/ml Doxycycline (+Dox) and then treated with different amounts of CX-5461. Cells were harvested one hour after addition of CX-5461 and the level of rRNA synthesis was determined by metabolic labelling as described [38]. Signals from CX-5461 treated cells were expressed as % from untreated cells (set as 100%), a line-of-best-fit was plotted and concentration of the drug inhibiting 50% of growth (GI_{50}) were calculated using GraphPad Prism 10. Standard deviations from 3 independent experiments are shown. (**C**–**E**) Quantitation of the (**C**) p-ATM, (**D**) p53, and (**E**) p-p53 blots from three independent experiments (Figure 2B). Error bars represent standard deviation (* $p < 0.05$, n = 3).

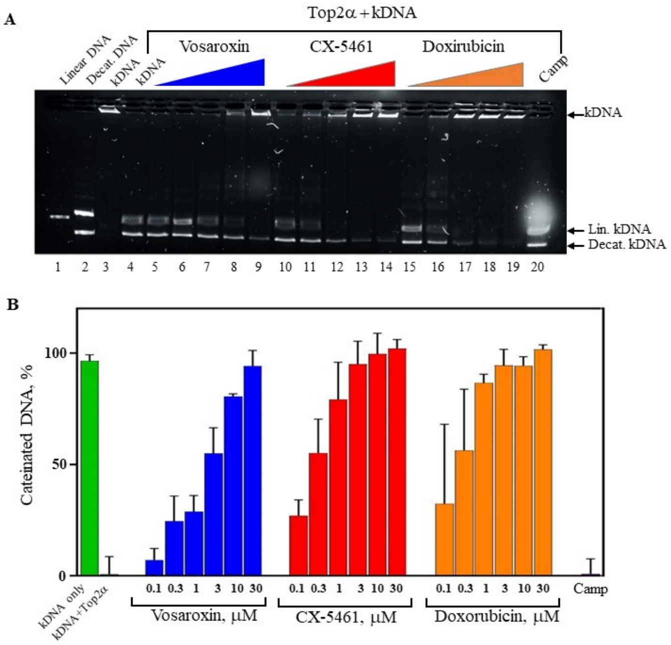

Figure S5. CX-5461 inhibits Top2α activity in decatenation assay. (**A**) Catenated kinetoplast DNA (kDNA) was incubated with recombinant Top2α in the presence of 0.1–10 μM drug for 30 min before undergoing agarose gel electrophoresis. The kDNA that was decatenated by Top2α appears as two bands as indicated by the decatenated DNA control. Top1 inhibitor camptothecin (Camp) is used as a negative control. Representative image is shown. (**B**) Quantification of results of the decatenation assay from three independent experiments. Signal from untreated kDNA was set as 100%. Error bars represent standard deviation, n = 3.

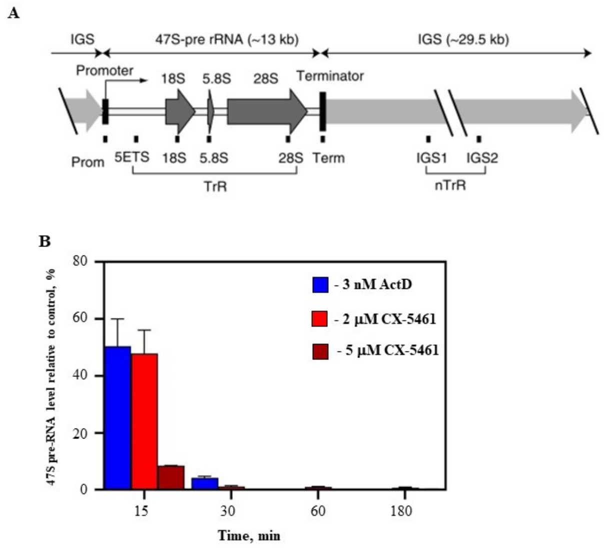

Figure S6. Doses Pol I inhibitors comparable with GI_{50} concentration rapidly inhibit rRNA synthesis in cells. (**A**) A diagram of the human rDNA repeat is shown indicating the positions of eight sets of specific PCR primer/probes used for qPCR analysis of immunoprecipitated DNA. ETS-external transcribed spacer; IGS, intergenic spacer; Prom, the rRNA promoter, term, the terminator. (**B**) HTETOP cells were treated with 3 nM of Actinomycin D, 2 μM and 5 μM of CX-5461 for various periods of time as indicated. The level of Pol I transcription was determined using qPCR and normalized to untreated control (set as 100%). Average values from three independent experiments were plotted as bar graph. Error bars represent standard deviation, n = 3.

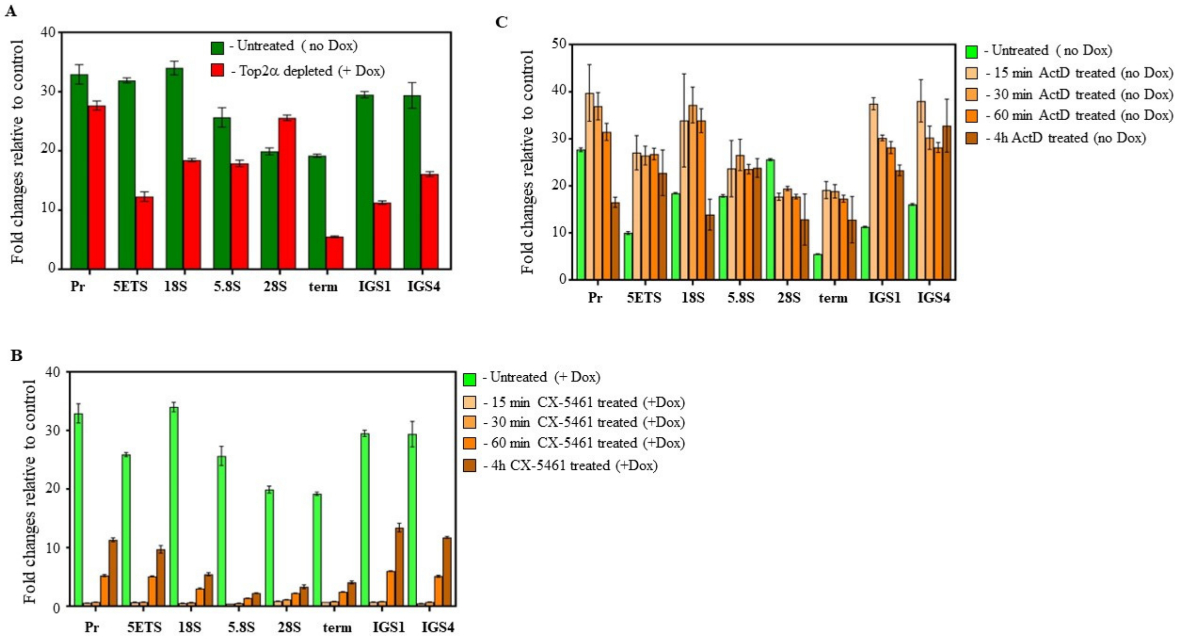

Figure S7. Control experiments confirming requirement of Top2α for formation of CX-5461 induced DSBs. (**A**) HTETOP cells were grown for 24 hours either untreated (no Dox) or in the presence of 1 mg/mL Doxycycline (+Dox). The DNA double-strand breaks (DSB) were labelled with BrdUTP using terminal transferase. DNA was extracted and sonicated to 400–600 bps fragments and the labelled DNA was enriched by anti-BrdUTP antibody. DNA enrichment was quantitated by qPCR. Signal was normalized to control (IgG) and plotted as fold changes. Error bars represent the standard deviation (n = 3). (**B**) HTETOP cells were grown the presence of 1 mg/ml Doxycycline (+Dox) and treated with 2 μM CX-5461 for various periods of time as indicated. Samples were processed and analyzed as in (A). Error bars represent the standard deviation (n = 3). (**C**) HTETOP cells were grown for 24 h untreated (no Dox) and then treated with 2 μM CX-5461 for various periods of time as indicated. Samples were processed and analyzed as in (**A**). Error bars represent the standard deviation (n = 3).

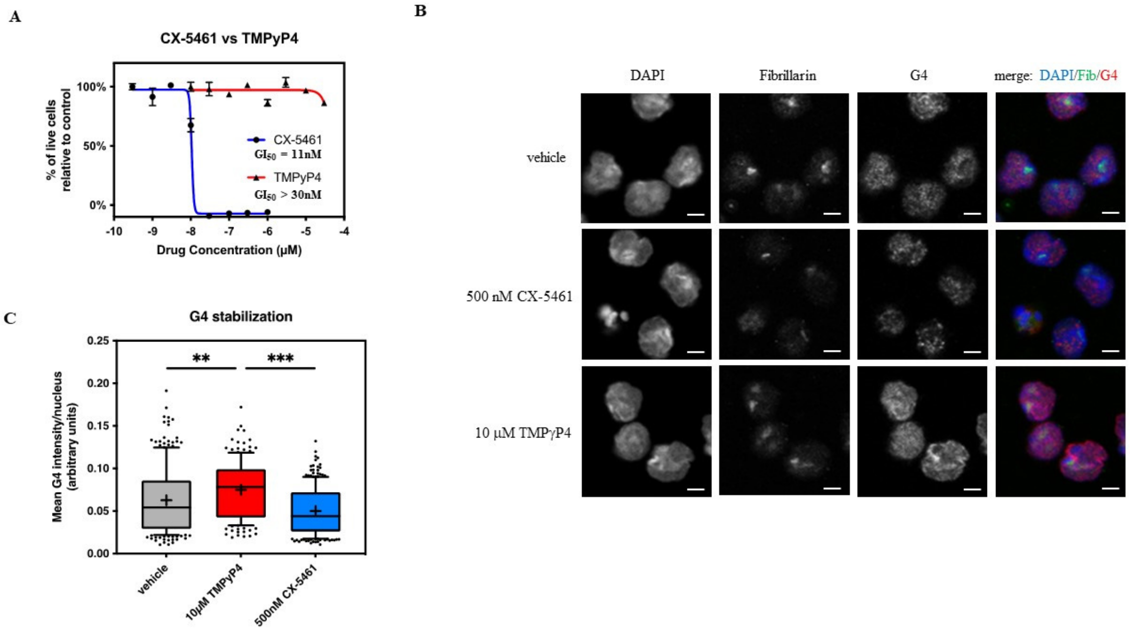

Figure S8. CX-5461 antitumor activity is not determined by G4 induction/stabilization. (**A**) *In vitro* dose response assay. T2A^{WT} cells were treated with either CX-5461 or TMPyP4 for 24 h in triplicate. Cell viability was measured by 570 nm absorption after Alamar blue treatment. Results were normalized by calibrating the readout from the vehicle-treated cells as 100% and the lowest readout as 0%, a line-of-best-fit was plotted and concentration of the drug inhibiting 50% of growth (GI_{50}) were calculated using GraphPad Prism 10. T2A^{WT} cell viability is not affected by TMPyP4 treatment at doses up to 30 µM. (**B**) Representative images of Eµ-*Myc* T2A^{WT} cells treated with vehicle, 500 nM CX-5461 or 10 µM TMPyP4 for 1 h and immunostained for the nuclei (DAPI), the nucleoli (Fibrillarin) and G-quadruplex DNA (G4). (**C**) Box plot of the quantitation of G4 DNA signal/nucleus from the experiment outlined in (**B**). Whiskers extend to the 10th and 90th percentile, mean represented by the plus sign (** $p < 0.01$; *** $p < 0.001$)
